# Pharmacotherapies for Diabetic Retinopathy: Present and Future

**DOI:** 10.1155/2007/52487

**Published:** 2007-06-28

**Authors:** Stephen G. Schwartz, Harry W. Flynn Jr.

**Affiliations:** ^1^Bascom Palmer Eye Institute, Retina Center at Naples, 311 9th Street North, Naples, FL 34102, USA; ^2^Bascom Palmer Eye Institute, 900 NW 17th Street, Miami, FL 33136, USA

## Abstract

Diabetic retinopathy remains a major cause of worldwide preventable blindness. Measures to avoid blindness include medical management (control of blood sugar, blood pressure, and serum lipids) and ocular management (laser photocoagulation and pars plana vitrectomy). Adjunctive pharmacologic therapies (intravitreal triamcinolone acetonide and anti-vascular endothelial growth factor agents) have shown early promise in the treatment of both diabetic macular edema and proliferative diabetic retinopathy. Other medications under investigation include the fluocinolone acetonide implantable device, extended-release dexamethasone implant, oral ruboxistaurin, and intravitreal hyaluronidase.

## 1. INTRODUCTION

Despite advancements in the delivery of ophthalmological care, diabetic retinopathy remains a major cause of preventable blindness [1]. The two most important visual complications of diabetic retinopathy are diabetic macular edema (DME) and proliferative diabetic retinopathy (PDR). Glycemic control [2, 3] and photocoagulation [4, 5] have been standard treatments for both DME and PDR for over 3 decades. Nevertheless, some patients suffer permanent visual loss despite prompt and appropriate therapy.

In recent years, further advances in pharmacotherapy have shown promise in the treatment of diabetic retinopathy. The three major classes of medications currently being studied are corticosteroids, vascular endothelial growth factor (VEGF) antagonists, and miscellaneous agents. Multiple clinical series have been reported (Table 1), and many more are ongoing or being planned (Table 2).

## 2. CORTICOSTEROIDS

**Figure 1:**
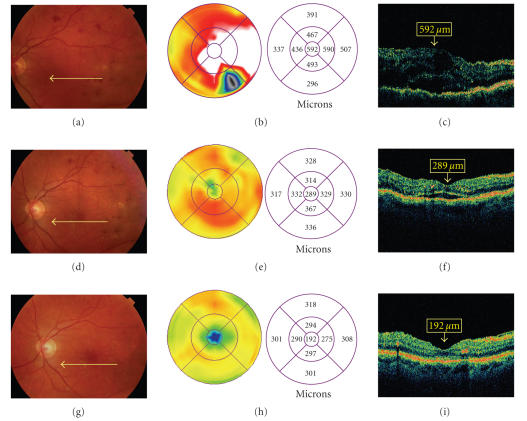
Intravitreal triamcinolone acetonide for diabetic macular edema. A patient presented with diabetic macular edema, visual acuity 20/60. Fundus photography (a) and optical coherence tomography (OCT) (b, c) are shown. The patient was treated with intravitreal triamcinolone acetonide. One month after treatment, visual acuity improved to 20/40, with improvement of macular edema on photography (d) and OCT (e, f). Four months after treatment, visual acuity improved to 20/20, with further improvement of macular edema on photography (g) and OCT (h, i).

Corticosteroids may work through multiple mechanisms of action. In addition to their well-known anti-inflammatory effects, corticosteroids may cause downregulation of VEGF [6, 7]. Intravitreal triamcinolone acetonide (IVTA) is commonly used today as an off-label adjunctive treatment of DME (Figure 1). Other intravitreal corticosteroids under study include a sustained-release device containing fluocinolone acetonide (Retisert; Bausch & Lomb, Rochester, NY) and extended-release dexamethasone in a biodegradable polymer (Posurdex, Allergan, Irvine, Calif).

### 2.1. Triamcinolone acetonide

Although intravitreal triamcinolone acetonide (Kenalog 40, Bristol-Myers Squibb, Princeton, NJ) has been administered for many years [8], its use has become more common since 2002 [9–11]. Recent prospective, randomized clinical trials have demonstrated generally favorable outcomes [12, 13]. The Diabetic Retinopathy Clinical Research network (DRCR.net) has completed enrollment on a three-year, randomized, prospective, multicenter clinical trial comparing two doses (1 mg and 4 mg) of preservative-free IVTA (Allergan, Irvine, Calif) with modified early treatment diabetic retinopathy study (ETDRS) photocoagulation for DME. Furthermore, IVTA may be a useful adjunct to photocoagulation for PDR, perhaps by decreasing the macular edema sometimes worsened by the treatment [14, 15].

The most important complication of IVTA is increased intraocular pressure (IOP) resulting in secondary open-angle glaucoma, which sometimes may be severe [16] and intractable [17, 18]. Elevation of IOP up to 24 mm Hg may occur in about 40% of patients, usually within about 3 months [19]. The second most important complication of IVTA is cataract formation, which may become visually significant in about half of eyes within 1 year [20].

The rates of injection-related endophthalmitis following IVTA have been reported to be in the range of 0.099%–0.87% per injection [21, 22]. The incidence of pseudoendophthalmitis, due to migration of triamcinolone acetonide crystals into the anterior chamber, is probably higher than that of infectious endophthalmitis. Other reported complications of IVTA (and of any intravitreal injection) include retinal detachment, lens trauma, and vitreous hemorrhage.

The use of peribulbar, rather than intravitreal, triamcinolone acetonide offers reduced risks of endophthalmitis and perhaps other complications. Peribulbar triamcinolone acetonide may have some limited efficacy for patients with DME [23, 24] although the bulk of the current literature appears to indicate that IVTA is more effective [25, 26]. DRCR.net has recently published a phase 2 randomized, prospective, multicenter clinical trial comparing peribulbar triamcinolone acetonide with and without photocoagulation. Peribulbar triamcinolone did not significantly improve vision in patients with mild DME [48]. Neither peribulbar triamcinolone nor IVTA appears to offer long-term efficacy for DME, which has led to the investigation of various extended-release corticosteroids.

### 2.2. Fluocinolone acetonide

The fluocinolone acetonide intravitreal implant (Retisert) is FDA-approved for the treatment of chronic, noninfectious posterior segment uveitis [27]. Although the device was also studied in patients with DME, no specific results has been published in the peer-reviewed literature at this time [28].

### 2.3. Extended-release dexamethasone

A bioerodable, extended-release dexamethasone implant (Posurdex, Allergan, Irvine, Calif) has shown favorable outcomes in the treatment of macular edema due to various etiologies, including DME, in a recent phase 2 study [29]. A phase 3 trial is underway.

## 3. VASCULAR ENDOTHELIAL GROWTH FACTOR (VEGF) INHIBITORS

Vascular endothelial growth factor (VEGF) increases retinal vascular permeability, causes breakdown of the blood-retinal barrier, and results in retinal edema [30]. VEGF is upregulated in diabetic retinopathy [31] and is present in increased levels in the aqueous and vitreous humor of patients with PDR [32, 33]. At least 5 isoforms of VEGF are known [34]. Three currently available anti-VEGF agents are pegaptanib, bevacizumab, and ranibizumab.

### 3.1. Pegaptanib

Pegaptanib (Macugen, OSI/Eyetech, Melville, NY) is a pegylated aptamer directed against the VEGF-A 165 isoform. It was the first FDA-approved ophthalmologic anti-VEGF agent, for the treatment of choroidal neovascularization from age-related macular degeneration (AMD) [35]. In a phase 2, prospective clinical trial, pegaptanib appeared to improve anatomic and visual outcomes in patients with DME [36]. Retrospective analysis of these data demonstrated some efficacy on retinal neovascularization as well [37]. Phase 3 trials of pegaptanib for DME are currently being conducted.

### 3.2. Bevacizumab

**Figure 2:**
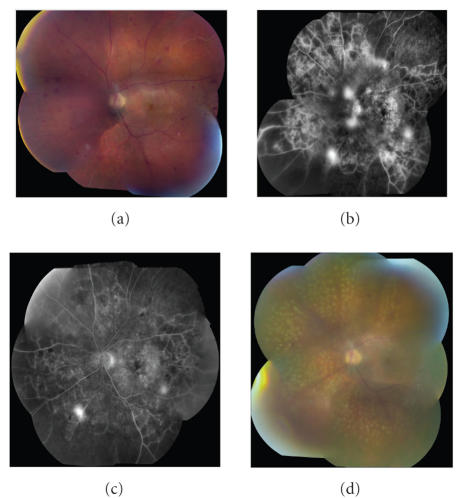
Intravitreal bevacizumab for proliferative diabetic retinopathy. A patient presented with proliferative diabetic retinopathy. Fundus photography (a) and fluorescein angiography (b) are shown. The patient was treated with intravitreal bevacizumab. Followup fluorescein angiography demonstrated improvement in angiographic leakage (c). Panretinal photocoagulation was then applied (d). (Case courtesy of Geeta Lalwani, MD, and Carmen A. Puliafito, MD, MBA.)

Bevacizumab (Avastin, Genentech, Inc., South San Francisco, Calif), a full-length recombinant humanized antibody, is active against all isoforms of VEGF-A. It is FDA-approved as an adjunctive systemic treatment for metastatic colorectal cancer [49]. Although off-label systemic bevacizumab has demonstrated some efficacy for exudative AMD [50], the agent has shown greater promise as an intravitreal medication. Case reports and small observational series have been reported using off-label intravitreal bevacizumab to treat exudative AMD [51], macular edema from nonischemic central retinal vein occlusion [52], iris neovascularization [53, 54], pseudophakic cystoid macular edema [55], and other diseases. Small, nonrandomized pilot studies have documented some efficacy against diffuse DME [56] and various complications of PDR [38–40] (Figure 2).

DRCR.net has completed enrollment on a phase 2, prospective, randomized, multicenter clinical trial to determine the safety and possible benefits of this agent. Plans for a phase 3 trial of two doses of an intravitreal anti-VEGF agent versus modified ETDRS grid laser photocoagulation for DME are under discussion.

### 3.3. Ranibizumab

Ranibizumab (Lucentis, Genentech, Inc., South San Francisco, Calif), a recombinant humanized antibody fragment, is active against all isoforms of VEGF-A. Intravitreal ranibizumab is FDA-approved for the treatment of exudative AMD [57, 58]. Two pilot studies of ranibizumab demonstrated some efficacy in the treatment of DME [41, 42]. DRCR.net is planning two phase 3, prospective, randomized, multicenter trials comparing patients. In the first trial, patients with DME and no PDR will be randomized to: (1) modified ETDRS grid laser photocoagulation; (2) photocoagulation before ranibizumab; (3) photocoagulation plus IVTA; or (4) ranibizumab before photocoagulation. In the second trial, patients with DME and PDR will be randomized to: (1) modified ETDRS grid laser photocoagulation plus scatter photocoagulation; (2) modified ETDRS grid laser photocoagulation plus scatter photocoagulation plus ranibizumab; or (3) modified ETDRS grid photocoagulation plus scatter photocoagulation plus IVTA.

The risk of injection-related endophthalmitis with the anti-VEGF agents is variable, but appears to be lower in more recent studies. Major prospective clinical trials of pegaptanib and ranibizumab reported rates between 0.7%–1.6% per eye [35, 57–59]. Most eyes in these reports received a series of injections, and a recent observational case series reported an incidence of 0.014% per injection for bevacizumab [60].

## 4. OTHER AGENTS

### 4.1. Ruboxistaurin

The enzyme protein kinase C *β* (PKC *β*) is activated by VEGF and appears to increase various systemic complications, including diabetic retinopathy [61]. Ruboxistaurin (Arxxant, Eli Lilly and Company, Indianapolis, Ind), an orally administered PKC *β* inhibitor, has shown efficacy against DME in two separate phase 3 trials [43, 44], although a recent study reported that treatment did not delay disease progression over a 30-month followup [45]. A smaller study noted that ruboxistaurin treatment was associated with a reduction in retinal vascular leakage, as measured by vitreous fluorometry, but visual acuity was not affected [46]. Although Lilly received an approvable letter from the FDA on August 18, 2006, the FDA requested an additional, 3-year, Phase 3 clinical trial to collect additional efficacy data in spite of an appeal with additional data.

### 4.2. Hyaluronidase

In an attempt at pharmacologic vitreolysis, intravitreal purified ovine hyaluronidase (Vitrase, ISTA Pharmaceuticals, Irvine, Calif) was proposed to accelerate clearance of vitreous hemorrhage from PDR and other causes. A recent phase 3 prospective clinical trial showed some favorable efficacy [47] and safety [62] outcomes for the clearance of vitreous hemorrhage due to all causes, but this agent is not currently FDA-approved for this indication.

## 5. CLINICAL GUIDELINES

Although none of the pharmacologic agents discussed above is FDA-approved for treatment of patients with diabetic retinopathy, off-label treatment can be considered for patients unresponsive to traditional standard care. These guidelines are summarized in Table 3.

In patients with diabetic macular edema not responsive to photocoagulation, either IVTA or an intravitreal anti-VEGF agent may be considered as second-line treatments. At this time, there are no published head-to-head comparisons of IVTA versus the anti-VEGF agents for this disease, although the pending DRCR.net trials may provide useful guidelines in this regard. Triamcinolone may be relatively more efficacious for DME, while the anti-VEGF agents appear more efficacious for PDR. Triamcinolone is considerably less expensive than the anti-VEGF agents, but is associated with risks of elevated IOP, cataract, and pseudoendophthalmitis.

In patients with complications of PDR not amenable to photocoagulation, intravitreal anti-VEGF agents may produce short-term stabilization or regression of iris and/or retinal neovascularization. In most patients, however, photocoagulation will eventually be necessary.

Intravitreal anti-VEGF agents may be helpful in patients with dense vitreous hemorrhage and patients with glaucoma secondary to neovascularization. If B-scan echography shows no evidence of retinal detachment, these agents may provide useful short-term anatomic improvement, until definitive photocoagulation can be given, or to reduce intraoperative bleeding in eyes with neovascular glaucoma. 

## 6. SUMMARY

Clinical experience with pharmacologic treatment for diabetic retinopathy continues to increase and reported outcomes in observational case series are promising. At this time, improved metabolic control and local ocular treatments (photocoagulation and vitrectomy) remain the proven treatments, through evidence-based medicine. As prospective randomized clinical trials accumulate data, the role of pharmacologic treatments will become clearer.

## Figures and Tables

**Table 1:** Selected published clinical series evaluating pharmacotherapies for advanced diabetic retinopathy. CMT: central macular thickness (assessed by optical coherence tomography), FA: fluorescein angiography, IVTA: intravitreal triamcinolone acetonide, DME: diabetic macular edema, NV: neovascularization, PDR: proliferative diabetic retinopathy, TA: triamcinolone acetonide, VA: visual acuity, and VH: vitreous hemorrhage.

Study (reference)	Reported patients	Reported eyes	Medication	Indication and outcome measures	Efficacy

Martidis et al. [9]	16	16	IVTA	DME, VA, CMT	Favorable
Gillies et al. [12]	43	69	IVTA	DME, VA	Favorable
Lam et al. [13]	63	63	IVTA	DME, VA, CMT	Favorable
Tunc et al. [23]	50	60	Peribulbar TA	DME, VA, FA	Favorable
Bakri et al. [24]	50	63	Peribulbar TA	DME, VA	Favorable
Cardillo et al. [25]	12	24	Peribulbar TA	DME, VA, CMT	Unfavorable
Bonini-Filho et al. [26]	36	36	Peribulbar TA	DME, VA, CMT	Unfavorable
Kuppermann et al. [29]	286	286	Extended-release dexamethasone	DME, VA, CMT	Favorable
Cunningham et al. [36]	172	172	Pegaptanib	DME, VA, CMT, photocoagulation	Favorable
Adamis et al. [37]	16	20	Pegaptanib	PDR, retinal NV	Favorable
Spaide et al. [38]	2	2	Bevacizumab	PDR, retinal NV, VH	Favorable
Jorge et al. [39]	15	15	Bevacizumab	PDR, VA, retinal NV, FA	Favorable
Avery et al. [40]	32	45	Bevacizumab	PDR, VA, iris and retinal NV, FA	Favorable
Chun et al. [41]	10	10	Ranibizumab	DME, VA, CMT	Favorable
Nguyen et al. [42]	10	10	Ranibizumab	DME, VA, CMT	Favorable
PKC-DRS [43]	252	504	Ruboxistaurin	DME, PDR, VA, Photocoagulation	Favorable
PKC-DRS2 [44]	685	1370	Ruboxistaurin	DME, VA, Photocoagulation	Favorable
PKC-DMES [45]	686	1372	Ruboxistaurin	DME, Photocoagulation	Unfavorable
Strom et al. [46]	41	55	Ruboxistaurin	DME, VA, vitreous fluorometry	Somewhat favorable
Kuppermann et al. [47]	1125	1125	Hyaluronidase	VH, anatomic clearance, VA	Favorable
DRCR.net et al. [48]	109	129	Peribulbar TA	DME, VA, CMT	Unfavorable

**Table 2:** Selected ongoing clinical trials evaluating pharmacotherapies for advanced diabetic retinopathy. DME: diabetic macular edema, DRCR.net: Diabetic Retinopathy Clinical Research network, PF/IVTA: preservative-free intravitreal triamcinolone acetonide, PDR: proliferative diabetic retinopathy, and TA: triamcinolone acetonide.

Sponsor	Medication	Indication	Status

DRCR.net (phase 3)	PF/IVTA	DME	Completed enrollment
Bausch & Lomb	Fluocinolone implant	DME	Completed enrollment
Allergan (phase 3)	Extended-release dexamethasone	DME	In planning stages
OSI/Eyetech	Pegaptanib	DME	Enrolling patients
DRCR.net (phase 2)	Bevacizumab	DME	Completed enrollment
Genentech	Ranibizumab	DME	Enrolling patients
DRCR.net (phase 3)	Ranibizumab or IVTA	DME with no PDR	Enrolling patients
DRCR.net (phase 3)	Ranibizumab or IVTA	DME with PDR	Enrolling patients

**Table 3:** Guidelines for pharmacologic treatment of advanced diabetic retinopathy.
